# Angiogenesis Inhibition in Prostate Cancer: An Update

**DOI:** 10.3390/cancers12092382

**Published:** 2020-08-23

**Authors:** Chandrani Sarkar, Sandeep Goswami, Sujit Basu, Debanjan Chakroborty

**Affiliations:** 1 Department of Pathology, Ohio State University, Columbus, OH 43210, USA; Chandrani.Sarkar@osumc.edu (C.S.); Sandeep.Goswami@osumc.edu (S.G.); Sujit.Basu@osumc.edu (S.B.); 2 Comprehensive Cancer Center, Ohio State University, Columbus, OH 43210, USA; 3 Department of Medical Oncology, Ohio State University, Columbus, OH 43210, USA

**Keywords:** prostate cancer, angiogenesis, angiogenic growth factors, antiangiogenic therapy

## Abstract

Prostate cancer (PCa), like all other solid tumors, relies on angiogenesis for growth, progression, and the dissemination of tumor cells to other parts of the body. Despite data from in vitro and in vivo preclinical studies, as well as human specimen studies indicating the crucial role played by angiogenesis in PCa, angiogenesis inhibition in clinical settings has not shown significant benefits to patients, thus challenging the inclusion and usefulness of antiangiogenic agents for the treatment of PCa. However, one of the apparent reasons why these antiangiogenic agents failed to meet expectations in PCa can be due to the choice of the antiangiogenic agents, because the majority of these drugs target vascular endothelial growth factor-A (VEGFA) and its receptors. The other relevant causes might be inappropriate drug combinations, the duration of treatment, and the method of endpoint determination. In this review, we will first discuss the role of angiogenesis in PCa growth and progression. We will then summarize the different angiogenic growth factors that influence PCa growth dynamics and review the outcomes of clinical trials conducted with antiangiogenic agents in PCa patients and, finally, critically assess the current status and fate of antiangiogenic therapy in this disease.

## 1. Introduction

Prostate cancer (PCa) is the most commonly diagnosed non-skin cancer in the United States and the second major cause of cancer-related death in men [1]. Although nearly 80% of cases are diagnosed as localized diseases that can be cured by radiotherapy or surgery, there is relapse of the disease in 30–60% of patients [2,3]. Androgen deprivation therapy (ADT) is commonly used in PCa treatment to block the androgens required for cancer growth [4,5,6]. However, aggressive disease relapse frequently occurs following ADT, and the disease becomes castration-resistant prostate cancer (CRPC) [4,5,6,7,8]. Treatment and management thus pose a real challenge at this stage of the disease [7,8]. Advancement of clinical research over the last two decades, along with the approval of several targeted and immunomodulatory agents, together with chemotherapeutic agents such as docetaxel, prednisone, and mitoxantrone, have substantially changed the treatment landscape of metastatic CRPC (mCRPC). Although these agents have shown significant benefits to a percentage of patients, these benefits are short-lived [9,10,11,12,13]. Accordingly, there is a constant need to identify newer and better treatments that can be used alone or in combination with currently available therapies for better disease management and outcomes.

Angiogenesis, the sprouting of new blood vessels from pre-existing vessels, is essential for tumor growth and metastasis [14,15,16,17]. Inhibiting angiogenesis has emerged as an effective strategy for the treatment of many solid tumors [18,19,20]. Unlike other angiogenic solid tumors, the inhibition of angiogenesis in PCa did not meet the clinical expectations, thereby igniting concerns about its relevance in PCa progression [21,22,23,24,25]. However, several preclinical observations as well as studies involving patient samples and cell lines support and reinforce the importance of angiogenesis in PCa [21,23,24,25]. Based on our current knowledge and relevant reports in literature, we will here discuss the importance of angiogenesis in PCa and the relevance of antiangiogenic strategies for the management of this disease.

## 2. Angiogenesis and Tumor Progression

Angiogenesis, a multistep process tightly regulated by several stimulatory and inhibitory growth factors, is essential not only for normal physiological processes but also for abnormal conditions, such as tumor growth [14,15,22,26]. Tumor growth often depends on its ability to sustain adequate blood supply by newly formed blood vessels (neovessels), thus making angiogenesis a rate-limiting step [14,15,16,26,27]. Vascular endothelial growth factors (VEGFs) are the most prominent and well-studied proangiogenic factors associated with tumor growth, including PCa [15,22,25]. Other angiogenic factors, such as fibroblast growth factors (FGFs), angiopoietins (Ang), hepatocyte growth factor (HGF), epidermal growth factor (EGF), platelet-derived growth factor (PDGF), placental growth factor (PlGF), insulin-like growth factors (IGFs), tumor necrosis factor (TNF), interleukin-6 (IL-6), and lysophosphatidic acid (LPA), have also been mentioned in the literature [22,28]. The antiangiogenic factors include angiostatin, endostatin, platelet factor-4 (PF4), tissue inhibitors of metalloproteinases (TIMPs), interleukins (ILs), and interferons (IFs) [22,25]. The neovessels formed during tumorigenesis differ from normal vasculature both in structure and in function, as they lack hierarchy, are chaotic in their arrangement, and the blood flow in them is sluggish [25,27,29,30]. 

Dr. Folkman, in his pioneer study, first described the essential role of angiogenesis in tumor growth in 1971, which led to the idea that targeting this process might be a promising therapeutic strategy to combat the growth and metastatic spread of cancer. Accordingly, antiangiogenic agents have been developed and tested in clinics, and many have been approved for the treatment of different cancer types [31]. 

## 3. Angiogenesis and Prostate Cancer 

Preclinical animal experiments and studies with clinical samples have indicated significant angiogenic activity within malignant prostate tumor tissues often measured as microvessel density (MVD), a well-established marker of angiogenesis, which correlated well with the tumor growth, Gleason score, and metastasis in PCa [23,25,32]. Thus, MVD in PCa has been designated as a valuable prognostic indicator that may predict the clinical and biochemical recurrence of the disease [23,25,32,33,34,35]. In addition, several reports have demonstrated the importance of angiogenic factors such as VEGFs, FGFs, ILs, transforming growth factor β (TGFβ), and different metalloproteinases which support the role of angiogenesis in PCa progression. However, the presently approved antiangiogenic agents could not meet acceptable outcomes in PCa patients, as was expected from the results of preclinical studies [23,24,25,36]. Although, in some cases, the response rate and progression-free survival were significantly improved, the overall survival (OS) did not increase following treatment [25]. Because there are discrepancies between these results and the reports from the preclinical animal studies and histopathology studies using PCa samples, it would be therefore prudent to discuss and analyze the possible reasons for these apparent differences. 

### 3.1. Angiogenic Growth Factors in Prostate Cancer

Vascular Endothelial Growth factors (VEGFs): VEGFs are part of the platelet-derived growth factor family and have been most extensively studied and described among all the angiogenic growth factors. VEGFs (VEGFA, VEGFB, VEGFC, and VEGFD) along with their cognate cell surface receptors (VEGFR1, VEGFR2, VEGFR3) play critical roles in PCa starting from cell growth to motility and cellular dissemination to other parts of the body [14,21,22]. Vascular endothelial growth factor –A (VEGFA), which is a 45 KDa heparin-binding protein, is the most predominant growth factor among all the VEGFs [14,22,37]. VEGFA has long been identified to influence every aspect of endothelial cell (EC) behavior and the maintenance of vascular integrity, and therefore plays a crucial role during tumor growth [14,17,22,37] and is overexpressed in PCa [37,38]. Both human PCa and PCa tissues of animal origin, including prostate tumors isolated from TRansgenic Adenocarcinoma Mouse Prostate (TRAMP)models, show an increased expression of VEGFA in comparison to normal prostate tissues [21,22,37,38,39,40,41]. Along with prostatic glandular epithelial cells, non-vascular cells of the tumor microenvironment, such as macrophages, fibroblasts, and mast cells, also secrete VEGFA [42,43]. The role of VEGFA in PCa progression is further evident from studies that correlate the increased VEGFA expression in PCa tissues to angiogenesis, advanced disease stages, increased recurrence, and decreased survival among patients [41,44,45]. Increased amounts of VEGFA are also present in the urine samples of PCa patients, which has been reported to serve as a prognostic indicator of hormone-refractory PCa progression and survivability of these patients [46,47]. In addition, the results from several preclinical studies indicate that VEGFA inhibition or treatment with anti-VEGFA antibodies blocks the growth of human prostate tumors through suppression of angiogenesis [21,22,48], further supporting the role of VEGFA-mediated angiogenesis in PCa growth and progression. Reports also show the efficacy of anti-VEGFA treatment in combination with other therapeutic agents in preclinical mouse PCa models [49,50]. Interestingly, a dose-dependent regulation in the expression of VEGFA and its receptor FLT1 (FMS-like tyrosine kinase receptor domain 1) or VEGFR1 by androgen during PCa progression has been reported [51]. The expression of VEGFR1 has been further correlated with higher MVD, advanced pathologic state, and poor outcome in PCa [51]. Moreover, patients with advanced PCa receiving ADT show genetic polymorphisms in the androgen receptor (AR) binding site of FLT1 [52,53]. These reports, therefore, indicate that the expression of VEGFA and its receptors in PCa are subjected to androgen regulation, which together regulates the process of angiogenesis. 

Fibroblast growth factors (FGFs): In addition to the VEGF family, the FGF family of growth factors is another major cytokine family that plays diverse roles during PCa progression [54,55]. FGFs are potent mitogens to many cell types, including ECs, and are expressed in many tissues, where they play significant roles in both physiological and pathological processes [54,55]. FGFs, particularly FGF2, FGF7, and FGF10, play vital roles in normal prostatic development, such as organogenesis, tissue homeostasis, and the acquisition of androgen dependency [54,55,56]. Both PCa cells and stromal cells in the PCa microenvironment secrete FGFs and express FGF receptors (FGFRs) [54,56,57]. FGF1 and FGF2 were among the first identified angiogenic factors which promote angiogenesis during tumor growth [57]. FGF/FGFR signaling regulates PCa angiogenesis both in a VEGFA-dependent and -independent manner [54,55,56,57,58,59]. Enhanced FGF levels and FGFR expressions such as type 1 FGFR (FGFR1), together with aberrant FGFR signaling and the loss of the intrinsic FGF7/FGF10-type 2 FGFR (FGFR2), are associated with enhanced PCa growth and angiogenesis [54,57,58]. The serum basic FGF (bFGF) level has also been shown to increase in PCa patients [59]. Furthermore, the correlation between FGF8 expression and VEGFA has been reported to be associated with advanced disease stage, higher serum PSA values, and poor survival [60]. These studies on the FGF/FGFR signaling cascade form the basis of FGF/VEGFR dual inhibition as a therapeutic strategy in PCa [61]. The prognostic implication of FGF, however, is controversial, as some studies have failed to find any relation between the FGF expression and PCa disease stage [62,63]. The specific role of FGF thus needs to be studied in more details.

Matrix metalloproteinases (MMPs): MMPs, together with TIMPs, form a classic regulatory unit that drives angiogenic processes both positively and negatively [64,65]. Metalloproteinases are zinc-containing calcium-dependent endopeptidases belonging to the metzincin superfamily [64,65]. Although MMPs are better known for their roles in tumor invasion and metastasis, as they help in breaking down the connective tissue barrier and thus help cancer cells to metastasize, they also play a crucial role in regulating angiogenesis by controlling EC attachment /detachment to the extracellular matrix, therefore helping in EC migration and invasion [64,65,66]. The expression of TIMPs controls the MMP activities in the tissue environment, and an imbalance in the expression of MMPs and TIMPs has been shown in PCa angiogenesis [64]. Studies reveal a higher MMPs to TIMPs ratio in advanced PCa tumors (Gleason score of 8 and above) compared to tumors with a favorable prognosis (Gleason score of less than 6) [67]. Knowledge about the involvement of MMPs in PCa progression is mostly derived from knockdown and overexpression studies using animal models of PCa due to the lack of availability of specific MMP inhibitors [68]. Among different members of the metalloproteinase family, the roles of MMP-2, -7, -9, and MT1-MMP are well documented in PCa. MMP-2, MMP-7, and MMP-9 have been shown to stimulate PCa angiogenesis, as conditional knockouts of these MMPs in mice resulted in PCa that showed reduced vascularity and angiogenesis [68]. While MMP-2 deficiency is associated with a reduction in the number of immature blood vessels along with reduced tumor burden, MMP-9 primarily plays a role in vascular remodeling [68]. Studies have further shown that knocking down of MMP-9 in PCa cells has a negative effect on the expression of proangiogenic molecules, such as VEGFA and intercellular adhesion molecule1 (ICAM-1). It helps in the upregulation of the expression of angiostatin and other endogenous inhibitors of angiogenesis in PCa [64,69,70,71]. Furthermore, not only in primary tumors, MMPs, specifically MMP-9, derived from osteoclasts directly affect angiogenesis in the prostate tumor-bone microenvironment [72]. However, controversies exist regarding the roles of MMPs in PCa progression, particularly that of MMP-9, as some studies correlate the increased expression of MMP-9 with a high Gleason score, disease progression, and poorer clinical outcomes [68,73]. On the contrary, others fail to demonstrate MMP-9 expression in PCa and describe the increased perivascular invasion of PCa cells in mice lacking MMP9 [68,73]. 

Transforming growth factor (TGF) β: TGFβ, a pleiotropic molecule comprised of three isoforms, exhibits potent tumor suppressor properties in the early stages of tumor development [74,75], while harboring a tumor-promoting effect during the later stages of tumor progression [75,76]. This paradoxical nature of TGFβ in PCa is mostly due to its capability to differentially activate the ERK/MAP kinase pathway in benign and malignant PCa cells [76]. Although no unanimous opinion exists regarding the time point when TGFβ switches from being a tumor suppressor to a tumor promoter, studies mostly report that it acts in the interphase of stromal -epithelial interaction and exerts its effect through three different TGFβ receptors—TGFβ1, TGFβ2, and TGFβ3on tumor cells, as well as on nonmalignant stromal cells such as fibroblasts and ECs [76]. Increased TGFβ1 in PCa tissues and high levels of TGFβ1 in the urinary and serum samples of PCa patients have been reported to be associated with enhanced angiogenesis, metastasis, and poor clinical outcomes [76,77]. TGFβ indirectly affects PCa angiogenesis via the upregulation of VEGFA through the activation of SMAD-mediated transcriptional regulation and activation of the Src/Focal Adhesion Kinase (FAK)/Protein kinase B (PKB or AKT) signaling pathways [78]. TGFβ also regulates PCa angiogenesis by promoting the differentiation of cancer-associated fibroblasts (CAFs), which in turn promote tumor angiogenesis through increased VEGFA production [79]. Besides this, VEGFA also influences TGFβ expression through a positive feedback mechanism [78]. However, some studies also report the negative association between TGFβ and VEGFA expression, especially in ECs [79]. Among the TGFβ receptors, TGFβ1 expression is associated with higher clinical tumor stages and a lower 5-year survival rate. Furthermore, apigenin, a natural flavone compound, and an inhibitor for TGFβ have been shown to inhibit angiogenesis in PCa through the suppression of VEGFA, which further proves the role of TGFβ in PCa angiogenesis [78].

Cyclooxygenases: Cyclooxygenases are enzymes that form prostaglandins and thromboxanes from arachidonic acids and are mainly associated with inflammatory responses [80,81]. Fatty acids and inflammation and their role in genitourinary cancer is an actively growing area of research [80,81]. Although clinical data at this point does not strongly support the effect of nonsteroidal anti-inflammatory drugs (NSAIDs) in inhibiting or preventing PCa progression in patients, the results from several preclinical studies are encouraging [80,81,82]. Cyclooxygenases and their eicosanoids products, prostaglandins and thromboxanes, play multiple roles in the regulation of EC biology [82]. There are two different forms of cyclooxygenase: cyclooxygenase 1 (COX1), which is expressed constitutively, and cyclooxygenase 2 (COX2), which expresses under the influence of various growth factors and cytokines [80,81]. The increased expression of COX2 has been reported in different cell types of the tumor microenvironment, and it promotes angiogenesis through enhanced VEGFA production, EC mobilization, vascular sprouting, and increased EC survival [82]. COX2 has been reported to overexpress in PCa tissues compared to the normal prostate, which shows low to no expression [83,84,85]. Increased COX2 expression is associated with increased MVDs in PCa tissues [84,85]. The inhibition of COX2 with its specific inhibitor, NS398, inhibits the growth of PC3 human prostate tumors in athymic mice through the suppression of neovessel formation in these tumors [85]. The inhibition of COX2 induces apoptosis in ECs via the suppression of AKT phosphorylation in PCa [86]. Importantly, epidemiologic studies show a lower risk of PCa in men taking aspirin and other NSAIDs, which has been attributed to COX2 inhibition that leads to the inhibition of subsequent angiogenesis [80,81]. However, specific patient data showing the grade-specific upregulation of COX2 in PCa is still lacking and is needed in order to ascertain the role of COX2 in PCa in a more definitive way. 

Interleukins (ILs): ILs, which are cytokines primarily secreted by leukocytes, play a major role in shaping the tumor microenvironment during tumor progression primarily through their immune regulatory properties [87,88]. In addition to lymphocytes, monocytes, and macrophages, ECs in the tumor microenvironment are regarded as major contributors of ILs [88]. To date, 50 different ILs have been identified [89]. ILs play diverse roles in PCa, such as being molecular determinants of progression from androgen-dependent to androgen-independent stages, or acting as tumor suppressors [90]. They regulate EC properties and angiogenesis in PCa either positively or negatively [91,92]. While some ILs, such as IL8, have been linked to increased PCa angiogenesis, others such as IL27 and IL10 have been linked to angiogenesis suppression in PCa [93,94,95]. IL8 expression in PCa has been shown to correlate with intra-tumoral MVD [93]. In addition, PCa cells transfected with IL8 have been shown to grow faster in mice with increased tumor vascularity compared to non-transfected cells [93]. As a result, targeting IL8 in PCa has emerged as a novel strategy for PCa treatment [93]. Other ILs such as IL27 and IL10, however, negatively regulate the process of angiogenesis during PCa progression. Rather than directly affecting ECs, the antiangiogenic properties of IL27 are mediated through the downregulation of proangiogenic-related genes such as FLT1, prostaglandin G/H synthase 1/cyclooxygenase-1 (PTGS1/COX-1), and FGFR3s and the upregulation of antiangiogenic genes such as CXCL10 and TIMP3 [94]. In addition to IL27, IL10 also negatively affects proangiogenic cells in the tumor microenvironment, such as activated macrophages, by inhibiting proangiogenic MMP2 and upregulating TIMP 1 and thereby suppressing the process of angiogenesis during PCa progression [95].

Other factors: In addition to the above factors, in recent years, there have been reports that other novel factors. such as microRNAs (miRNAs), which are short segments (21- to 25-nucleotides) of non-coding RNAs; long noncoding RNAs (lncRNAs), which are RNA transcripts longer than 200 nucleotides that do not encode proteins; and extracellular vesicles (EVs), which are small cell-derived membranous structures containing proteins, lipids, and genetic material, either directly or indirectly affect the angiogenic response in PCa [96,97,98,99,100,101,102]. In the past decades, these factors have attracted attention, as they play important roles in the progression of the disease. Increasing evidence indicates that cancer cells communicate among themselves as well as with cells of the surrounding microenvironment via the secretion and transfer of these factors. We will discuss some of these recent findings. 

miRNAs can modulate the functions of ECs via non-cell-autonomous as well as cell-autonomous mechanisms, and thus regulate angiogenesis [103]. They regulate the expressions of both pro- or anti-angiogenic growth factors, and target the growth factor receptors and signaling molecules required in the process. Both the upregulation and downregulation of miRNAs have impact on PCa progression and angiogenesis. While miRNAs such as miR-296, miR-30d, miR-323, miR-21, and miR-182 are upregulated in PCa [104,105,106,107,108,109], the decreased expressions of miR-195, miR-218, and miR-146a are also shown to be associated with increased angiogenesis in PCa [97,110,111,112]. The upregulation of miR-30d [104] and miR-323 [105] were reported to enhance VEGF synthesis and secretion by PCa cells and therefore promote VEGF-mediated angiogenesis in PCa. miR-296, which is frequently upregulated in PCa, regulates the levels of VEGF and PDGF receptors in angiogenic ECs [108,109]. miR-21 and miR-182 regulate the expression of HIF1α and thereby HIF 1α-mediated angiogenesis [106,107]. The decreased expression of miR-146a was reported in CRPC, where it regulates the expression of epidermal growth factor receptor (EGFR) and MMP2 in PCa tissues [112]. On the other hand, the decreased expression of miR-195 in PCa results in the upregulation of ribosomal protein S6 kinase B1 (RPS6KB1), which leads to increased expressions of MMP-9 and VEGF proteins, which regulate angiogenesis [110]. miR-218, which inhibits angiogenesis through targeting the rapamycin-insensitive companion of mTOR (RICTOR)/VEGFA axis, is also downregulated during PCa progression [97]. In addition, the reduced expression of miR-130b in PCa tissues correlates with poor prognosis and increased angiogenesis, as the miR-130b/TNF-α/NF-κB/VEGFA loop inhibits PCa angiogenesis [113].

Among the lncRNAs, prostate cancer antigens (PCAs) and prostate cancer-associated transcripts (PCAT) are of immense interest, as they regulate several aspects of PCa progression. PCA3, a prostate-specific RNA which is overexpressed in more than 95% of PCa patients’ urine samples, has been reported to regulate the expression of genes involved in angiogenesis, in addition to genes controlling signal transduction and apoptosis [114,115]. The knockdown of lncRNas, PCAT3, and PCAT9 in PCa cells leads to the suppression of VEGF synthesis and angiogenesis via the modulation of the miR-203/SNAI2 axis [116]. RBMS3-AS3, which poorly expresses in PCa, can suppress PCa angiogenesis and cell proliferation by upregulating the expression of an intrinsic angiogenesis inhibitor, vasohibin1 (VASH1), through the RBMS3-AS3/miR-4534/VASH1 axis [117]. 

EVs, including apoptotic bodies, microvesicles, and exosomes, play vital roles in vascular development, growth, and maturation [118]. EVs can act both in a positive and negative way to modulate the process of tumor growth and angiogenesis, therefore they are considered as promising targets for therapeutic intervention [102,119,120]. While exosomes secreted from PCa cells, cells in the PCa microenvironment, and also from PCa stem cells mostly promote angiogenesis, exosomes derived from other cell types and tissues have been reported to negatively affect the process and thereby PCa growth, suggesting a crucial role of EVs in tumor angiogenesis, which largely depends on their origin [102,120,121]. Cancer-derived EVs bestow aggressive phenotypes to cancer cells by affecting ECs within the tumor microenvironment and promoting angiogenesis. The exosomes in bodily fluids, secreted during hypoxia or acidocis, cause increased angiogenesis [122]. EVs contain miRNAs, mRNAs, and proteins that mediate the communication between various cell types and ECs and induce either pro- or antiangiogenic signaling. Sphingomyelin transferred into ECs by EVs secreted by PCa cells promotes the migration and proangiogenic activity of these cells [123]. Exosomes from PCa cell lines contain TGFβ1, which stimulates the differentiation of fibroblasts to highly aggressive myofibroblasts [124,125], an important source of matrix-remodeling proteins within the tumor microenvironment, via the activation of TGFβ/SMAD3 signaling, and thereby support PCa angiogenesis [124,125]. PCa cell-derived exosomes promote the differentiation of mesenchymal stem cells (MSCs) to proangiogenic myofibroblasts that support angiogenesis during PCa progression [126]. Furthermore, PCa-associated exosomes contain c-Src, IGF-1R, and FAK proteins that promote angiogenesis and PCa development [127]. Prostate-specific membrane antigen (PSMA), which is an important tumor marker for PCa progression, including angiogenesis and metastasis, is enriched in exosomes derived from PCa cells [128].

**Figure 1:**
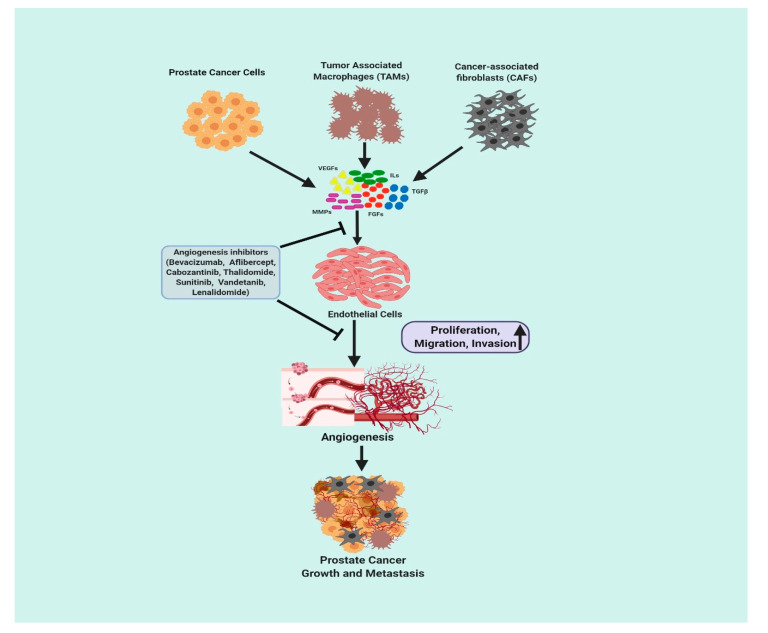
Schematic diagram of the role of angiogenic growth factors in prostate cancer (created with BioRender.com https://biorender.com/). VEGFs = Vascular endothelial growth factors; ILs = Interleukins, MMPs = Matrix metalloproteinases; FGFs = Fibroblast growth factors; TGFβ = Transforming growth factor beta.

A schematic diagram representing the role of angiogenic growth factors in PCa is presented in Figure 1.

### 3.2. Current Antiangiogenic Treatment Strategies for Prostate Cancer

Several mechanisms, such as inhibiting the activity of the proangiogenic factors directly, blocking the receptors of these proangiogenic factors, or elevating the levels of endogenous antiangiogenic factors, can be employed to inhibit angiogenesis [24,25]. In PCa, strategies were mainly designed to inhibit the proangiogenic factors or target downstream signaling effector pathways using monoclonal antibodies or small molecule inhibitors or using agents that are capable of immune modulation. Due to the multitude of factors regulating the process of angiogenesis, monotherapy, as well as combination therapy with different chemotherapeutic agents or antiangiogenic agents, have been tested for optimal therapeutic effects. Since VEGFA is a critical growth factor associated with PCa, it has been extensively studied [25,41,44,45]. As the overexpression of VEGFA correlates with poor prognosis and metastasis, the main antiangiogenic strategies in PCa at present were designed to mainly inhibit the VEGF pathway by targeting VEGFA or its receptors [25,41,44,45]. In this section, we will discuss some of these prominent antiangiogenic strategies that were developed for the treatment of PCa.

Bevacizumab is a humanized anti-VEGF monoclonal IgG1 antibody (molecular weight, 149 kDa) that selectively binds to and neutralizes VEGF, thereby preventing it from binding to its cell surface VEGFRs, leading to reduced MVD in tumors, thus limiting the blood supply to tumor tissues and lowering interstitial tissue pressure and vascular permeability [25]. A Phase II trial with bevacizumab in combination with ADT consisting of 102 recurrent hormone-sensitive PCa patients reported a significant improvement in relapse-free survival (RFS). Hypertension was the most commonly observed adverse effect in these patients [129]. Other Phase II studies with CRPC patients where bevacizumab was combined with docetaxel, thalidomide, and prednisone [130] or where bevacizumab was combined with estramustine and docetaxel for the treatment of HRPC or CRPC patients [131,132] have all demonstrated that the combination with bevacizumab was tolerated and led to encouraging antitumor activity, median survival and OS. On the contrary, the Phase III Cancer and Leukemia Group B (CALGB)trial that followed with 1050 metastatic PCa patients demonstrated some improvement in progression-free survival (PFS) with the combination therapy; there was no significant increase in OS. Bevacizumab also showed other adverse events (AE), which included cardiovascular and neutropenic complications [133]. Furthermore, in a very recent Phase I/II trial in patients with mCRPC, bevacizumab when used in combination with temsirolimus showed limited clinical activity, and only a transient decrease in the circulating tumor cells (CTC) level was observed, which was associated with significant AE [134]. These studies thus indicate that the addition of bevacizumab to standard therapy does not result in any significant clinical benefit in CRPC. 

Sunitinib is a novel oral small-molecule tyrosine kinase inhibitor that targets VEGFR1 and VEGFR2. [135]. Not many clinical studies have been conducted using sunitinib in PCa. In a randomized, placebo-controlled, Phase III trial conducted with 873 progressive mCRPC patients who either received prednisone in combination with sunitinib or prednisone alone, sunitinib did not improve OS and severe AE was reported, which led to the discontinuation of the study [136].

Vandetanib is an oral multi-tyrosine kinase inhibitor that targets VEGFR2, epidermal growth factor receptor (EGFR), and RET (rearranged during transfection) pathways in cancer [25]. In a randomized, double-blinded, placebo-controlled Phase II trial of vandetanib in combination with docetaxel/prednisolone in 86 hormone-refractory PCa patients, the combination with vandetanib did not demonstrate any benefit [137]. Additionally, in another randomized Phase II trial with mCRPC patients, a combination of vandetanib with bicalutamide did not exhibit superior efficacy compared to the treatment with bicalutamide alone. These approaches were also associated with considerable toxicity [138].

Aflibercept (VEGF Trap) is a recombinant human fusion protein comprised of extracellular domains of human VEGFR1 and 2 fused to the constant region (Fc) of human immunoglobulin G1 (IgG1), which has a very high VEGF binding affinity and binds to all isomers of the VEGFA and B family and PlGF [25]. In a Phase III double-blinded randomized trial, where men with mCRPC received aflibercept with docetaxel and prednisone as first-line chemotherapy, no improvement in OS was reported. Furthermore, a high incidence of severe AE and treatment-related fatal events were reported in the aflibercept group compared to the placebo group [139]. 

Thalidomide is an oral agent that inhibits the activity of angiogenic factors such as VEGF, bFGF, and IL-6. In CRPC patients who have failed multiple therapies, thalidomide monotherapy showed some clinical activity [140]. Results from an open-label Phase II trial of thalidomide in patients with androgen-independent PCa [141] indicated thalidomide to be an option for patients who do not respond to other forms of therapy. Upon combination with docetaxel in a randomized Phase II trial, more than half (53%) of the CRPC patients had a PSA decrease of at least 50%, as compared to 35% of the patients in the docetaxel-alone treatment arm [142]. 

Lenalidomide is a thalidomide derivative that inhibits VEGF-mediated phosphatidylinositol-3,4,5-trisphosphate (PI3K)-Akt signaling pathway. The results of an open-label, Phase II clinical trial with 63 CRPC patients where lenalidomide was combined with docetaxel, bevacizumab, and prednisone showed that combining different angiogenesis inhibitors was safe with appropriate supportive measures and could potentially provide clinical benefit to patients [143]. In a Phase I/II double-blinded, randomized study with 60 non-metastatic PCa patients, treatment with lenalidomide showed an acceptable toxicity, with disease stabilization and reduction in PSA [144]. However, in a randomized, double-blind, placebo-controlled, Phase III study with 1059 chemotherapy-naive mCRPC patients, a combination of lenalidomide with docetaxel and prednisone resulted in a significantly worse OS with increased AE such as hematological side effects, diarrhea, pulmonary embolism, and asthenia [145]. 

Cabozantinib is an orally available small-molecule inhibitor of kinases, including VEGFR2. Preclinical studies show that cabozantinib can effectively inhibit PCa growth and metastasis by suppressing angiogenesis [146]. Clinical studies conducted so far with cabozantinib have demonstrated positive effects mostly in context to bone metastasis inhibition, bone lesion resolution, and improvement in patient CTC counts [25]. In a Phase II randomized trial of cabozantinib with patients with advanced solid tumors, randomization was halted, and the patients were unblinded because the drug showed efficacy and the largest PFS improvement in CRPC patients [147]. Furthermore, cabozantinib treatment also reduced soft tissue and bone lesions, bone turnover markers, pain, and narcotic use [148]. However, in a Phase III study with previously treated mCRPC patients, cabozantinib did not improve the OS, disease progression, or PSA response [149].

From the results of the clinical studies that have been summarized in Table 1, it can be concluded that the antiangiogenic approach in PCa has only been moderately successful. Treatment-related toxicities, often grade 3 or greater, were observed with these agents, which also resulted in treatment-related deaths. The main AE reported were hypertension, gastrointestinal perforation, proteinuria, hemorrhage, thrombosis, fistula formation, cardiac toxicity, endocrine dysfunction, and reversible posterior leukoencephalopathy [25,150].

## 4. Conclusions and Future Direction

In summary, there is substantial evidence regarding the critical role of angiogenesis in the progression of PCa, with studies reporting correlations between expressions of angiogenic markers, Gleason scores, metastatic disease progression, and clinical outcomes [23,25,32,33,34,35]. Several studies have also demonstrated the efficacies of antiangiogenic agents in preclinical PCa models [23,25]. However, despite these promising results, antiangiogenic treatment has only been moderately successful in some hormone-sensitive PCa patients [96]. On the contrary, in mCRPC, which to date has limited treatment options, antiangiogenic treatment has failed to show any significant effects in terms of improvement in OS or improvement in the quality of life of patients in the clinics [21,25].

Reviews on PCa and angiogenesis have discussed many factors that may be responsible for the moderate effectiveness of antiangiogenic agents in PCa [22,151,152]. Importantly, the failure of these agents may be attributed to several factors, such as differences in the design of clinical trials from preclinical studies, choosing appropriate angiogenic agents or combination of agents, the determination of treatment response and endpoints, and lastly the side effects encountered by the patients [23,25]. 

Treatment response measured as a decrease in the PSA level, improvement in PFS, or OS may not also be sufficient and appropriate for antiangiogenic agents, as the PSA level may not always accurately indicate the clinical status and disease progression, and these drugs often increase the PSA level despite a positive disease response [22,23,25]. The measurement of alternative biomarkers such as CTCcounts in the peripheral blood samples isolated from patients can be an indicator of drug efficacy as CTC counts can predict OS better than PSA levels at all time points [153,154]. OS as an endpoint may not be the best evaluator of survival benefit from an antiangiogenic agent, considering the long survival rate of PCa patients normally, and PFS is not an ideal endpoint to determine the efficacy of a drug, as it may or may not necessarily translate into an OS improvement [23]. Therefore, new biomarkers of disease progression and the establishment of clinical endpoints following the administration of antiangiogenic agents may help in the determination of the efficacy of these drugs. Furthermore, at present there are no established markers to assess the angiogenic activity in PCa. MVD, which is considered as a potent surrogate marker, may not be an independent prognostic factor in untreated tumors, and studies have not yet established a strong correlation between MVD and the effectiveness of antiangiogenic agents in PCa [155]. The determination of vascularization in two-dimensional histological slides may not also be the most appropriate method for evaluating the efficacy of antiangiogenic agents [155,156,157]. With improved imaging techniques and other noninvasive techniques such as Doppler, it will be prudent to assess the whole vascular architecture within the tumors. Moreover, the study of the functional aspects of angiogenesis, such as the detection of vascular permeability and blood flow in tumors, will help to provide previously unavailable information and also help in decision-making [29,156,157].

Finally, although several growth factors regulate angiogenesis in PCa, most of the preclinical studies and clinical trials have been undertaken with anti-VEGFA or anti-VEGFR agents, which demonstrated modest clinical response and severe AE in patients. Therefore, it will be necessary to investigate the roles of other novel proangiogenic growth factors in PCa, which will identify newer, effective, and safe antiangiogenic agents for the treatment of PCa. Resistance, both intrinsic and acquired, to the currently used antiangiogenic agents is another possible reason for the suboptimal performance of these agents in the clinics. A number of growth factors can activate different signaling pathways during the process of angiogenesis in PCa. Recent findings indicate the probable regulatory roles of miRNAs, lncRNAs, and EVs in PCa angiogenesis. Therefore, targeting the angiogenic process using agents that are capable of inhibiting multiple pathways or by combining agents that can target different pathways may help to overcome drug resistance and result in better clinical outcomes. Combination therapy with antiangiogenic agents have actually shown promising results in clinics [143,151]. Furthermore, to find out more effective drug combinations and minimize toxicity, detailed studies determining the effective dose of each drug in a combination and monitoring the pharmacodynamic endpoints is required. Importantly, a deeper understanding of the process of angiogenesis and signaling pathways regulating the process is needed in order to design novel targeted antiangiogenic therapies in PCa.

## Figures and Tables

**Table 1:** Summary of clinical studies of antiangiogenic drugs in the treatment of prostate cancer.

Drug	Mechanism of Action	Phase	Drugs in Combination Treatment	Outcomes	Adverse Events	Reference
*Bevacizumab* (Avastin®; Genentech, Inc.)	Recombinant humanized monoclonal antibody against VEGF-A	I/II	Temsirolimus	Transient decrease in circulating tumor cell levels in metastatic CRPC patients	Anorexia Fatigue Mucositis Lymphopenia Thrombocytopenia	[134]
		II	ADT	Significant improvement in relapse-free survival in recurrent hormone-sensitive PCa patients	Hypertension Musculoskeletal pain Infection Headache	[129]
			Docetaxel, Thalidomide, and Prednisone	Improved median survival in CRPC patients	Neutropenia Anemia Thrombocytopenia Constipation	[130]
			Estramustine and Docetaxel	Improved overall survival in CRPC patients	Leukopenia Neutropenia Fatigue Pulmonary embolism Deep venous thrombosis Epistaxis Hypertension	[132]
		III	Docetaxel and Prednisone	No improvement in overall survival in metastatic CRPC patients	Neutropenia Fatigue Leucopenia Hypertension GI hemorrhage and perforation Mucositis	[133]
*Sunitinib malate* (Sutent®, Pfizer)	Novel oral small-molecule tyrosine kinase inhibitor that targets VEGFR1 and VEGFR2	III	Prednisone	No improvement in overall survival in metastatic CRPC patients	Diarrhea Nausea Vomiting Fatigue Hand/Foot Syndrome Hypertension Mucosal inflammation Asthenia	[136]
*Vandetanib* (Caprelsa, AstraZeneca & Sanofi)	Oral multi-tyrosine kinase inhibitor that targets VEGFR2, epidermal growth factor receptor (EGFR), and RET pathways in cancer	II	Docetaxel and Prednisolone	No efficacy benefit in HRPC patients	Fatigue Diarrhea Nausea Erythematous and exfoliative rash	[137]
			Bicalutamide	No efficacy benefit in metastatic CRPC patients	Hypertension Fatigue Diarrhea Dyspnea Skin rash Hand/Foot syndrome Anorexia Prolonged QTc interval	[138]
*Aflibercept*/VEGF Trap (Eylea and Zaltrap, Regeneron Pharmaceuticals)	Recombinant human fusion protein that has high VEGF binding affinity and binds to all isomers of the VEGFA and B family and placental growth factor (PGF)	III	Docetaxel and Prednisone	No improvement in overall survival in metastatic CRPC patients	Hypertension Vascular Disorder GI hemorrhage Epistaxis Perforation Stomatitis Ulceration	[139]
*Thalidomide* (Thalomid, Celgene)	Oral agent that inhibits VEGF, bFGF, and IL-6	II	Docetaxel	PSA decrease in CRPC patients	Thrombocytopenia Anemia Venous thromboembolism	[142]
*Lenalidomide* (Revlimid, Celgene)	Thalidomide derivative that inhibits VEGF-mediated phosphatidylinositol-3,4,5-trisphosphate (PI3K)-Akt signaling pathway	I/II		Disease stabilization and reduction of PSA in non-metastatic PCa patients	Neutropenia Venous thromboembolism Fatigue Hyperglycemia Constipation Anemia	[144]
		II	Docetaxel, Bevacizumab, and Prednisone	PSA decline and partial responses in CRPC patients	Neutropenia Anemia Thrombocytopenia Diarrhea Fatigue	[143]
		III	Docetaxel and Prednisone	Significantly worse overall survival in chemotherapy-naive metastatic CRPC patients	Hematological side effects Diarrhea Pulmonary embolism Asthenia Pneumonia	[145]
*Cabozantinib* (Cometriq^TM^ and Cabometyx^TM^, Exelixis Inc.)	Orally available small-molecule inhibitor of kinases, including VEGFR2	II		Proliferation-free survival improvement in CRPC patients	Fatigue Diarrhea Hypertension Muscle spasms Asthenia	[147]
		III		No improvement in overall survival, disease progression, or PSA response in previously treated metastatic CRPC patients. Some improvement in bone scan response, radiographic progression-free survival, symptomatic skeletal events, circulating tumor cell conversion, and bone biomarkers	Nausea Diarrhea Fatigue Asthenia Weight loss Constipation Anemia Hypertension Bone pain	[149]
